# Direct cost of trabeculectomy in the Brazilian public health system: a micro-costing analysis in a university hospital*

**DOI:** 10.1590/1980-220X-REEUSP-2026-0293en

**Published:** 2026-08-31

**Authors:** Joice Alves Cabral, Priscila de Castro Handem, Ana Paula Rodrigues Siqueira, Giovanni Nicola Umberto Italiano Colombini, Daniel Aragão Machado, Carlos Roberto Lyra da Silva, Alexander Itria, Thiago Louro, Andrea Alves de Andrade, Roberto Carlos Lyra da Silva

**Affiliations:** 1 Universidade Federal do Estado do Rio de Janeiro, Hospital Universitário Gaffrée e Guinle, Rio de Janeiro, RJ, Brazil.; 2 Universidade Federal do Estado do Rio de Janeiro, Escola de Enfermagem Alfredo Pinto, Rio de Janeiro, RJ, Brazil.; 3 Instituto Nacional do Câncer, Programa de Pós-Graduação em Oncologia, Rio de Janeiro, RJ, Brazil.; 4 Universidade Federal de São Carlos, São Carlos, SP, Brazil.; 5 Universidade Federal Fluminense, Rio das Ostras, RJ, Brazil.; 6 Universidade Federal do Rio de Janeiro, Centro de Ciências Matemáticas e da Natureza, Rio de Janeiro, RJ, Brazil.

**Keywords:** Glaucoma, Costs and Cost Analysis, Ophthalmologic Surgical Procedures, Unified Health System, Perioperative Nursing.

## Abstract

**Objective:**

To estimate the direct cost of trabeculectomy in a university hospital within the Brazilian Public Health System (SUS) and compare it to the reimbursement for the procedure.

**Method:**

Documentary, retrospective, and quantitative study, with micro-costing based on Activity-Based Costing. Sixty-seven eligible medical records from 76 trabeculectomies performed between 2022 and 2024 were analyzed. Surgical materials and intraoperative medications were considered.

**Results:**

The average direct cost was R$ 388.32 per procedure, while the average amount actually billed to the SUS was R$ 602.25 per procedure. Suture threads accounted for 45% of the total cost, followed by intraoperative medications (28%) and consumables (27%). Persistence of the surgical waiting list and an increase in regulated waiting times were observed.

**Conclusion:**

The direct cost of trabeculectomy was lower than the reimbursement from the SUS; however, the economic viability of the procedure did not automatically translate into timely access.

## INTRODUCTIOn

Glaucoma is a progressive optic neuropathy characterized by structural changes in the optic disc and retinal nerve fiber layer, often associated with elevated intraocular pressure (IOP). Considered a major cause of irreversible blindness worldwide, glaucoma imposes a significant social and economic burden on healthcare systems, especially in middle-income countries like Brazil^(1,2)^. Within the Brazilian Public Health System (SUS), managing this condition requires a balance between early diagnosis, continuous pharmacological therapy, and timely surgical intervention^(3)^.

Trabeculectomy (TREC) has remained, since its introduction, the “gold standard” surgical technique for controlling IOP in cases where clinical treatment with eye drops is insufficient or unfeasible. By creating a fistula that allows aqueous humor to drain into the subconjunctival space, TREC aims to stabilize the visual field and prevent progression to amaurosis.

Despite the emergence of new technologies, such as drainage implants and minimally invasive surgeries, trabeculectomy remains relevant due to its long-term effectiveness and potential for lower operational costs for the public healthcare system^(4)^. However, the Brazilian public health scenario is marked by funding constraints and uncertainties regarding the sufficiency of the amounts reimbursed by the Management System of the SUS Table of Procedures, Medications, Orthotics, Prosthetics and Special Materials (SIGTAP) to cover the actual costs of supplies, especially in university hospitals, which combine healthcare, teaching, and research functions.

The lack of accurate estimates of the direct cost of procedures compromises management decisions and can limit the provision of essential services. In the case of trabeculectomy, understanding the relationship between the cost of supplies, reimbursement from the SUS, and access through the regulatory system is fundamental to distinguishing financing problems from those related to the organization of care and the productive capacity of the service.

This study was designed to support hospital management and perioperative nursing by producing empirical evidence on direct costs, procedure sustainability, and organizational barriers to surgical access. The research question was: what is the actual direct cost of performing a trabeculectomy? Given that the scenario is a leading federal university hospital in ophthalmology, located in the Southeast region of Brazil, how does this value relate to reimbursement from the SUS and access via the regulatory system?

Health Technology Assessment aims to provide evidence to support decisions regarding the incorporation and use of procedures. Nevertheless, in the context of hospital management, micro-costing methods remain essential for identifying item- by-item consumption per patient and producing more accurate estimates of the direct cost of care, in contrast to aggregated macro-costing approaches^(5)^.

## METHOD

This is a quantitative, descriptive, documentary, and retrospective study, applying micro-costing from a hospital perspective, based on the *Activity-Based Costing* (ABC) method. The setting was a reference federal university hospital in ophthalmology located in the southeastern region of Brazil, within the SUS. The time frame included surgeries performed between 2022 and 2024.

The sample consisted of 67 medical records with complete data, derived from 76 trabeculectomies identified in this period. Patients who underwent primary trabeculectomy were included, with complete records of materials, medications, surgical time, age, sex, and billed amount. Combined surgeries and medical records with incomplete data that prevented the estimation of the direct cost of the procedure were excluded.

Data collection was performed using surgical schedules, paper-based medical records, billing sheets, and institutional records of materials and medications. Demographic and healthcare (age, sex, and surgical time), economic (supplies consumed, unit cost, and total direct cost), and billing (amount reimbursed by the SUS per procedure) variables were extracted. The monetary unit used was the Brazilian Real in the year 2025.

The reimbursement amounts considered in this study corresponded to the amounts actually billed and approved by the SUS for each procedure, as recorded in the billing sheets of the analyzed medical records. Therefore, the values presented reflect hospital production carried out between 2022 and 2024, and not just the nominal value in effect in the SIGTAP table for a specific period. Although the nominal value shown in the SIGTAP table is fixed per period, the values analyzed correspond to the amounts actually approved and recorded in the billing sheets of the medical records, subject to the processing rules in effect in each period.

Micro-costing was implemented in three stages: identification of the supplies used in each surgery, valuation of the items based on institutional acquisition records, and comparison of the calculated costs with the amounts actually billed to the SUS. Surgical materials and intraoperative medications directly related to performing trabeculectomy were considered. Indirect costs, such as human resources, energy, sterilization, equipment depreciation, and administrative expenses, were not included.

The total direct cost per surgery was estimated by summing the unit costs multiplied by the quantities consumed in each procedure, according to the expression: total direct cost = Σ (item quantity × item unit cost). Data were organized into electronic spreadsheets and analyzed using descriptive statistics, with calculation of mean, median, standard deviation, and annual distribution of procedures, costs, and reimbursements. Ethical Aspects: The study followed the ethical principles of Resolution No. 466/2012, using anonymized primary data and approval by a Research Ethics Committee, under opinion number 7.196.235^(6,7)^.

## RESULTS

The analysis of 67 eligible medical records, from a total of 76 surgeries performed between 2022 and 2024, allowed for a more precise characterization of the care and economic profile of trabeculectomy in the investigated scenario.

Seventeen trabeculectomies were performed in 2022, 35 in 2023, and 24 in 2024. From a demographic and clinical standpoint, the cohort presented a mean age of 59.09 years (standard deviation: 24.87), with a median of 66.5 years, evidencing an asymmetrical distribution and concentration of cases in older age groups, even with the presence of younger patients. The average surgery time was 1 hour and 46 minutes (standard deviation: 37 minutes and 8 seconds). The annual values showed a slight increase in the average direct cost per procedure over the series, from R$ 351.73 in 2022 to R$ 394.00 in 2023 and R$ 404.30 in 2024. Total reimbursements from the SUS remained higher than direct costs in all years analyzed, resulting in a favorable average difference of R$ 213.93 per procedure (Table 1).

**Table 1:** Annual comparison between reimbursement from the SUS and the direct cost of trabeculectomy – Rio de Janeiro, RJ, Brazil, 2022–20241.

Year	Number of procedures	Total SUS reimbursement (R$)	Total direct cost (R$)	Direct cost per procedure (R$)
2022	17	13,149.02	5,979.43	351.73
2023	35	26,725.30	13,789.85	394.00
2024	24	14,929.05	7,681.76	404.30

Source: Research data.

The breakdown of the average direct cost per procedure, estimated at R$ 388.32, showed a concentration of expenses in items critical to the safe technical execution of trabeculectomy, with a predominance of suture threads (45.0%), followed by intraoperative medications (28.0%), and consumable materials (27.0%). This distribution shows a dependence on specialized inputs and little room for cost reduction without potentially impacting the quality of care (Table 2).

**Table 2:** Composition of the average direct cost of trabeculectomy, according to supply category – Rio de Janeiro, RJ, Brazil, 2022–2024.

Supply category	Average cost (R$)	Representativeness (%)
Suture threads (nylon 10-0 and 8-0)	174.74	45.0
Intraoperative medications (mitomycin and anesthetics)	108.73	28.0
Consumable materials (blades, gloves, gauze and drapes)	104.85	27.0
**Total**	**388.32**	**100.0**

Source: Research data.

Access indicators showed a mismatch between the procedure’s sustainability potential and the system’s responsiveness. The average waiting time in the SISREG system increased from 18 days in 2020 to 122 days in 2025, in parallel with the persistence of unmet demand exceeding the service’s annual surgical production capacity.

## DISCUSSION

The results of this study provide a relevant interpretation for the debate on the sustainability of surgical care for glaucoma within the SUS. The identification of an average direct cost lower than the amount reimbursed by the SUS suggests, in principle, the economic viability of trabeculectomy from the perspective of measured supplies, in line with economic analyses already applied to the field of glaucoma^(4,7)^.

Previous studies on glaucoma indicate that trabeculectomy remains a highly effective and widely used procedure, particularly in settings with budget constraints. Furthermore, while newer surgical technologies offer promising therapeutic alternatives for selected cases, they may entail higher costs and more limited availability within public healthcare systems. In this context, trabeculectomy remains a relevant option for the sustainability of care.

However, this finding does not justify a simplistic interpretation of sufficient funding, since the micro-costing adopted exclusively covered intraoperative materials and medications, without including indirect and structural components involved in the delivery of care^(5)^. Thus, the main analytical contribution of this study lies in demonstrating that the relationship between cost and reimbursement should be interpreted in conjunction with installed capacity, the organization of the work process, and the effectiveness of surgical access. It should be noted that the comparison was made based on the amounts actually invoiced by the institution during the period studied, and not exclusively on the nominal values in effect in a single period of the SIGTAP table, which should be considered when interpreting the results.

Recent international literature reinforces the relevance of this interpretation. Economic model derived from *Treatment of Advanced Glaucoma Study* demonstrated that, in patients with advanced primary open-angle glaucoma, trabeculectomy is more likely to be cost-effective over the lifetime compared to purely medical treatment, with incremental gains in quality of life and a cost-per-QALY ratio considered acceptable in public health systems^(8)^.

Although this type of analysis relies on a different time horizon and cost structure than those used in the present study, its results converge in supporting the idea that filtration surgery should not be interpreted merely as an immediate increase in expenditure, but as an intervention potentially capable of reducing disease progression, the need for multiple therapies, and future costs associated with visual impairment.

This discussion becomes even more relevant when considering the clinical, social, and economic burden of glaucoma. Contemporary studies show that disease progression is associated with a decline in quality of life, functional impairment, increased frequency of anxiety and depression, and higher direct and indirect costs as visual loss worsens^(9,10)^.

Updates on the global burden of disease also indicate that glaucoma persists among the most significant causes of years lived with vision-related disability, especially in aging populations and in settings of unequal access to eye care. In this context, the analysis of isolated surgical costs has to be situated within a broader perspective of the disease trajectory, in which therapeutic delays can shift the system towards stages of greater clinical complexity and higher accumulated social costs^(11,12)^.

From an access perspective, the literature has also drawn attention to the impact of delays between surgical indication and actual entry into the operating room. A recent multicenter study on glaucoma filtration surgeries identified a median waiting time of 44 days between indication and procedure, highlighting that the preoperative phase often accounts for the largest share of the delay in care^(13)^.

Although the contexts of care organization are distinct, this finding aligns with the present study by highlighting that waiting time is a concrete component of clinical risk and organizational inefficiency. Even when delays do not immediately result in blindness or severe visual impairment, long-term follow-up evidence indicates greater visual field loss and faster progression when treatment is delayed, reinforcing the centrality of timely access as a relevant healthcare outcome^(14)^.

The persistent prolonged waiting time for the procedure reinforces the idea that the central problem is not limited to the direct cost of the surgery, but to the system’s inability to ensure timely and clinically appropriate treatment. In glaucoma, delays in treatment can result in irreversible progression of vision loss, with broad functional, social, and economic repercussions. From this perspective, the surgical waiting list ceases to be merely an administrative indicator and becomes a marker of healthcare risk, with potential impact on disability, dependence, and future need for rehabilitation and social support. The interpretation of the results, therefore, must consider not only the cost of the procedure itself, but also the cost of not providing timely specialized care.

In the field of perioperative nursing, the findings are particularly relevant as they demonstrate that skilled management of supplies, safe preparation of the surgical environment, and streamlined operating room turnover can contribute to greater efficiency without compromising the quality of care. In university hospitals, where healthcare, teaching, and research coexist, predictability in the consumption of materials and medications is strategic for care planning and for reducing avoidable cancellations. Thus, nursing occupies a central position in translating economic evidence into organizational practices capable of increasing surgical productivity and improving the institutional response to unmet demand.

The literature on perioperative management offers additional elements for interpreting this scenario. A Brazilian integrative review demonstrated that strategies based on Lean and Six Sigma can optimize patient flow, workflow, and performance indicators in the perioperative period, promoting greater operational stability and identifying avoidable bottlenecks^(15)^.

In parallel, an international systematic review on operating room turnaround time showed that modifiable measures, such as parallel processing, standardization of preparation, coordination between teams, and workflow reorganization, are consistently associated with improved operating room efficiency^(16)^.

In the context of ophthalmological services, where the volume of procedures can be highly sensitive to cumulative delays, these findings suggest that marginal organizational gains can have a significant impact on annual service capacity. In this regard, activity-based costing methods and their time-oriented variations have been described as strategic tools for bridging the gap between economic analysis and care management, by making visible the real cost drivers throughout the care journey^(17,18)^.

In teaching hospitals, this approach is particularly useful because it allows one to distinguish between costs inherent to the procedure, costs associated with operational inefficiency, and costs arising from institutional complexity. More than just measuring expenses, micro-costing can support decisions about standardizing surgical kits, tracking consumption, negotiating purchases, designing processes, and prioritizing improvement interventions. For nursing and hospital management, this means shifting the debate from purely financial control to the production of healthcare value, linking safety, productivity, and sustainability.

In terms of network management, the results suggest that expanding surgical services depends less on an immediate review of reimbursement values and more on strategies for care coordination, surgical scheduling, and strengthening of regulation. The coexistence of the procedure’s economic viability and the persistence of a significant waiting list indicates that organizational barriers may be producing allocative inefficiency, insofar as a potentially sustainable procedure does not translate into sufficient coverage. This finding is particularly relevant for public eye health policies, as it highlights the need to integrate economic evaluation, production monitoring, and access management as inseparable dimensions of the healthcare response to glaucoma.

The results should be interpreted in light of certain limitations. The analysis focused on a single institution and considered only direct costs, which limits the generalizability of the findings and prevents a more comprehensive estimate of the total hospital cost of trabeculectomy. Furthermore, the use of paper-based medical records and secondary records can introduce informational losses and limits the exploration of more detailed clinical variables. Nevertheless, the study provides relevant empirical evidence by documenting, based on real data, the gap between the economic sustainability of the procedure and the service’s actual capacity to respond, an aspect that has been little explored in the national literature.

Another limitation relates to the lack of transparency in the composition of the components used to define the Ambulatory Procedure Authorization reimbursement value, which restricts direct comparisons between the costs identified by micro-costing and the criteria used by the SUS financing system.

## CONCLUSION

This study demonstrated that, when considering the measured direct costs, trabeculectomy performed at a federal university hospital, reference in ophthalmology, located in the Southeast region of Brazil, had an average cost lower than the amount reimbursed by the SUS. This result indicates the economic and operational viability of the procedure from the perspective of intraoperative materials and medications, but it does not exhaust the analysis of institutional sustainability, since indirect and structural costs were not considered.

Meanwhile, the continued significant waiting list for the procedure demonstrated that the economic viability of the direct cost does not automatically translate into timely access, pointing to the influence of organizational, regulatory, and installed capacity factors on the healthcare response to glaucoma. The findings reinforce the usefulness of micro-costing as a tool to support hospital management and perioperative nursing, by allowing greater visibility into the consumption of supplies and supporting strategies for care planning, organization of surgical flow, and qualification of resource use within the context of the SUS.

## Data Availability

As advocated by Open Science, this is the repository where the research data has been deposited in its entirety. Repository name: http://www.repositorio-bc.unirio.br/. URL or DOI: http://hdl.handle.net/unirio/14982. Date of deposit: 2026-04-02.
