# Supplementary material for: Direct cost of trabeculectomy in the Brazilian public health system: a micro-costing analysis in a university hospital
Source: Rev Esc Enferm USP. 2026 Aug 31;60:e20260293. doi: 10.1590/1980-220X-REEUSP-2026-0293en (PMC13574318; doi:10.1590/1980-220X-REEUSP-2026-0293en)
Supplement: Translation

# Translation: Custo direto da trabeculectomia no Sistema Único de Saúde: análise de microcusteio em hospital universitário*

**DOI:** 10.1590/1980-220X-REEUSP-2026-0293pt

**Authors:** Joice Alves Cabral, Priscila de Castro Handem, Ana Paula Rodrigues Siqueira, Giovanni Nicola Umberto Italiano Colombini, Daniel Aragão Machado, Carlos Roberto Lyra da Silva, Alexander Itria, Thiago Louro, Andrea Alves de Andrade, Roberto Carlos Lyra da Silva

**Affiliations:** 1 Universidade Federal do Estado do Rio de Janeiro, Hospital Universitário Gaffrée e Guinle, Rio de Janeiro, RJ, Brasil.; 2 Universidade Federal do Estado do Rio de Janeiro, Escola de Enfermagem Alfredo Pinto, Rio de Janeiro, RJ, Brasil.; 3 Instituto Nacional do Câncer, Programa de Pós-Graduação em Oncologia, Rio de Janeiro, RJ, Brasil.; 4 Universidade Federal de São Carlos, São Carlos, SP, Brasil.; 5 Universidade Federal Fluminense, Rio das Ostras, RJ, Brasil.; 6 Universidade Federal do Rio de Janeiro, Centro de Ciências Matemáticas e da Natureza, Rio de Janeiro, RJ, Brasil.

**Keywords:** Glaucoma, Custos e Análise de Custo, Procedimentos Cirúrgicos Oftalmológicos, Sistema Único de Saúde, Enfermagem Perioperatória.

## Abstract

**Objetivo:**

Estimar o custo direto da trabeculectomia em hospital universitário do Sistema Único de Saúde e compará-lo ao reembolso do procedimento.

**Método:**

Estudo documental, retrospectivo e quantitativo, com microcusteio baseado em Activity-Based Costing. Foram analisados 67 prontuários elegíveis de 76 trabeculectomias realizadas entre 2022 e 2024. Consideraram-se materiais cirúrgicos e medicamentos intraoperatórios.

**Resultados:**

O custo direto médio foi de R$ 388,32 por procedimento, enquanto o valor médio efetivamente faturado ao SUS foi de R$ 602,25 por procedimento. Fios de sutura representaram 45% do custo total, seguidos por medicamentos intraoperatórios (28%) e materiais de consumo (27%). Observou-se persistência de fila cirúrgica e aumento do tempo de espera regulado.

**Conclusão:**

A trabeculectomia apresentou custo direto inferior ao reembolso do SUS; entretanto, a viabilidade econômica do procedimento não se converteu automaticamente em acesso oportuno.

## INTRODUÇÃO

O glaucoma é uma neuropatia óptica progressiva, caracterizada por alterações estruturais no disco óptico e na camada de fibras nervosas da retina, frequentemente associada a níveis elevados de pressão intraocular (PIO). Considerado importante causa de cegueira irreversível no mundo, o glaucoma impõe expressivo ônus social e econômico aos sistemas de saúde, especialmente em países de renda média, como o Brasil^(1,2)^. No âmbito do Sistema Único de Saúde (SUS), a gestão dessa condição exige equilíbrio entre diagnóstico precoce, terapia farmacológica contínua e intervenção cirúrgica oportuna^(3)^.

A trabeculectomia (TREC) permanece, desde a sua introdução, como a técnica cirúrgica “padrão-ouro” para o controle da PIO em casos em que o tratamento clínico com colírios é insuficiente ou inviável. Por meio da criação de uma fístula que permite o escoamento do humor aquoso para o espaço subconjuntival, a TREC visa estabilizar o campo visual e prevenir a progressão para a amaurose.

Apesar do surgimento de novas tecnologias, como os implantes de drenagem e as cirurgias minimamente invasivas, a trabeculectomia mantém relevância pela eficácia em longo prazo e pelo potencial de menor custo operacional para a rede pública^(4)^. No entanto, o cenário da saúde pública brasileira é marcado por restrições de financiamento e por incertezas quanto à suficiência dos valores reembolsados pelo Sistema de Gerenciamento da Tabela de Procedimentos, Medicamentos, Órteses, Próteses e Materiais Especiais do SUS (SIGTAP) para cobrir os custos reais dos insumos, especialmente em hospitais universitários, que acumulam funções assistenciais, de ensino e de pesquisa.

A ausência de estimativas precisas do custo direto dos procedimentos compromete decisões gerenciais e pode limitar a oferta de serviços essenciais. No caso da trabeculectomia, conhecer a relação entre custo dos insumos, reembolso do SUS e o acesso via sistema de regulação é fundamental para distinguir problemas de financiamento daqueles relacionados à organização do cuidado e à capacidade produtiva do serviço.

Este estudo foi delineado para subsidiar a gestão hospitalar e a enfermagem perioperatória, ao produzir evidência empírica sobre custo direto, sustentabilidade do procedimento e barreiras organizacionais ao acesso cirúrgico. A pergunta de pesquisa foi: qual o custo direto real da trabeculectomia realizada? Sendo o cenário um hospital universitário federal de referência em oftalmologia, localizado na região Sudeste do Brasil, como esse valor se comporta em relação ao reembolso do SUS e ao acesso via sistema de regulação?

A Avaliação de Tecnologias em Saúde busca oferecer evidências para decisões sobre incorporação e uso de procedimentos. No plano da gestão hospitalar, entretanto, métodos de microcusteio permanecem essenciais para identificar o consumo item a item por paciente e produzir estimativas mais acuradas do custo direto do cuidado, em contraste com abordagens agregadas de macrocusteio^(5)^.

## MÉTODO

Trata-se de estudo quantitativo, descritivo, documental e retrospectivo, com aplicação de microcusteio na perspectiva hospitalar, fundamentado no método *Activity-Based Costing* (ABC). O cenário foi um hospital universitário federal de referência em oftalmologia localizado na região Sudeste do Brasil, no Sistema Único de Saúde. O recorte temporal compreendeu cirurgias realizadas entre 2022 e 2024.

A amostra foi composta por 67 prontuários com dados completos, provenientes de 76 trabeculectomias identificadas no período. Foram incluídos pacientes submetidos à trabeculectomia primária, com registro completo de materiais, medicamentos, tempo cirúrgico, idade, sexo e valor faturado. Excluíram-se cirurgias combinadas e prontuários com incompletude de dados que impedisse a estimação do custo direto do procedimento.

A coleta de dados foi realizada a partir do mapa cirúrgico, prontuários físicos, folhas de faturamento e registros institucionais de materiais e medicamentos. Foram extraídas variáveis demográficas e assistenciais (idade, sexo e tempo cirúrgico), variáveis econômicas (insumos consumidos, custo unitário e custo total direto) e variáveis de faturamento (valor reembolsado pelo SUS por procedimento). A unidade monetária utilizada foi o Real Brasileiro no ano de 2025.

Os valores de reembolso considerados neste estudo corresponderam aos montantes efetivamente faturados e aprovados pelo SUS para cada procedimento, conforme registro nas folhas de faturamento dos prontuários analisados. Dessa forma, os valores apresentados refletem a produção hospitalar realizada entre 2022 e 2024 e não apenas o valor nominal vigente na tabela SIGTAP em competência específica. Embora o valor nominal constante na tabela SIGTAP seja fixo por competência, os valores analisados corresponderam aos montantes efetivamente aprovados e registrados nas folhas de faturamento dos prontuários, sujeitos às regras de processamento vigentes em cada período.

O microcusteio foi operacionalizado em três etapas: identificação dos insumos utilizados em cada cirurgia, valoração dos itens a partir de registros institucionais de aquisição e confronto dos custos apurados com os valores efetivamente faturados ao SUS. Foram considerados materiais cirúrgicos e medicamentos intraoperatórios diretamente relacionados à execução da trabeculectomia. Não foram incluídos custos indiretos, como recursos humanos, energia, esterilização, depreciação de equipamentos e despesas administrativas.

O custo direto total por cirurgia foi estimado pela soma dos custos unitários multiplicados pelas quantidades consumidas em cada procedimento, conforme a expressão: custo direto total = Σ (quantidade do item × custo unitário do item). Os dados foram organizados em planilhas eletrônicas e analisados por estatística descritiva, com cálculo de média, mediana, desvio-padrão e distribuição anual dos procedimentos, custos e reembolsos. Aspectos Éticos: O estudo observou os princípios éticos da Resolução nº 466/2012, com uso de dados primários anonimizados e aprovação por Comitê de Ética em Pesquisa, sob parecer nº 7.196.235^(6,7)^.

## RESULTADOS

A análise dos 67 prontuários elegíveis, oriundos de um total de 76 cirurgias realizadas entre 2022 e 2024, permitiu caracterizar com maior precisão o perfil assistencial e econômico da trabeculectomia no cenário investigado.

Foram realizadas 17 trabeculectomias em 2022, 35 em 2023 e 24 em 2024. Do ponto de vista demográfico e clínico, a coorte apresentou média de idade de 59,09 anos (desvio-padrão: 24,87), com mediana de 66,5 anos, evidenciando distribuição assimétrica e concentração de casos em faixas etárias mais avançadas, ainda que com presença de pacientes mais jovens. O tempo médio de cirurgia foi de 1h46min (desvio-padrão: 37min08s. Os valores anuais mostraram discreta elevação do custo direto médio por procedimento ao longo da série, de R$ 351,73 em 2022 para R$ 394,00 em 2023 e R$ 404,30 em 2024. Os reembolsos totais do SUS permaneceram superiores aos custos diretos em todos os anos analisados, resultando em diferença média favorável de R$ 213,93 por procedimento (Tabela 1).

**Tabela 1:** Comparação anual entre reembolso do SUS e custo direto da trabeculectomia – Rio de Janeiro, RJ, Brasil, 2022–20241.

Ano	Número de procedimentos	Reembolso SUS total (R$)	Custo direto total (R$)	Custo direto por procedimento (R$)
2022	17	13.149,02	5.979,43	351,73
2023	35	26.725,30	13.789,85	394,00
2024	24	14.929,05	7.681,76	404,30

Fonte: Dados da pesquisa.

A decomposição do custo direto médio por procedimento, estimado em R$ 388,32, evidenciou concentração dos gastos em itens críticos à execução técnica segura da trabeculectomia, com predominância dos fios de sutura (45,0%), seguidos por medicamentos intraoperatórios (28,0%) e materiais de consumo (27,0%). Essa distribuição mostra dependência de insumos especializados e baixa margem para redução de custos sem possível repercussão sobre a qualidade assistencial (Tabela 2).

**Tabela 2:** Composição do custo direto médio da trabeculectomia, segundo categoria de insumo – Rio de Janeiro, RJ, Brasil, 2022–2024.

Categoria de insumo	Custo médio (R$)	Representatividade (%)
Fios de sutura (nylon 10-0 e 8-0)	174,74	45,0
Medicamentos intraoperatórios (mitomicina e anestésicos)	108,73	28,0
Materiais de consumo (lâminas, luvas, gazes e campos)	104,85	27,0
**Total**	**388,32**	**100,0**

Fonte: Dados da pesquisa.

Os indicadores de acesso mostraram descompasso entre o potencial de sustentabilidade do procedimento e a capacidade de resposta do sistema. O tempo médio de espera no SISREG aumentou de 18 dias, em 2020, para 122 dias, em 2025, em paralelo à persistência de demanda reprimida superior à capacidade anual de produção cirúrgica do serviço.

## DISCUSSÃO

Os resultados deste estudo permitem uma interpretação relevante para o debate sobre sustentabilidade da atenção cirúrgica ao glaucoma no âmbito do Sistema Único de Saúde. A identificação de custo direto médio inferior ao valor reembolsado pelo SUS sugere, em princípio, viabilidade econômica da trabeculectomia sob a ótica dos insumos mensurados, em consonância com análises econômicas já aplicadas ao campo do glaucoma^(4,7)^.

Estudos prévios sobre glaucoma indicam que a trabeculectomia permanece como um procedimento de elevada efetividade clínica e ampla utilização, especialmente em cenários de restrição orçamentária. Além disso, tecnologias cirúrgicas mais recentes, embora representem alternativas terapêuticas promissoras para casos selecionados, podem apresentar maior custo e disponibilidade mais limitada em sistemas públicos de saúde. Nesse contexto, a trabeculectomia permanece como uma alternativa relevante para a sustentabilidade assistencial.

No entanto, esse achado não autoriza uma leitura simplificada de suficiência de financiamento, uma vez que o microcusteio adotado contemplou exclusivamente materiais e medicamentos intraoperatórios, sem incluir componentes indiretos e estruturais que participam da produção do cuidado^(5)^. Assim, a principal contribuição analítica deste estudo reside em demonstrar que a relação entre custo e reembolso deve ser interpretada em articulação com a capacidade instalada, a organização do processo de trabalho e a efetividade do acesso cirúrgico. Ressalta-se que a comparação foi realizada com base nos valores efetivamente faturados pela instituição no período estudado, e não exclusivamente nos valores nominais vigentes em uma única competência da tabela SIGTAP, o que deve ser considerado na interpretação dos resultados.

A literatura internacional recente reforça a pertinência dessa interpretação. Modelo econômico derivado do *Treatment of Advanced Glaucoma Study* demonstrou que, em pacientes com glaucoma primário de ângulo aberto avançado, a trabeculectomia apresenta maior probabilidade de custo-efetividade ao longo da vida quando comparada ao tratamento exclusivamente medicamentoso, com ganho incremental de qualidade de vida e razão de custo por QALY considerada aceitável em sistemas públicos de saúde^(8)^.

Embora esse tipo de análise se apoie em horizonte temporal e estrutura de custos diferentes daqueles empregados no presente estudo, seus resultados convergem ao sustentar que a cirurgia filtrante não deve ser interpretada apenas como incremento de gasto imediato, mas como intervenção potencialmente capaz de reduzir progressão da doença, necessidade de múltiplas terapias e custos futuros associados à deficiência visual.

Essa discussão torna-se ainda mais relevante quando se considera a carga clínica, social e econômica do glaucoma. Estudos contemporâneos mostram que a progressão da doença está associada a piora da qualidade de vida, comprometimento funcional, maior frequência de ansiedade e depressão e incremento de custos diretos e indiretos à medida que a perda visual se agrava^(9,10)^.

Atualizações da carga global da doença também indicam persistência do glaucoma entre as causas mais relevantes de anos vividos com incapacidade relacionados à visão, especialmente em populações envelhecidas e em cenários de desigualdade de acesso à atenção oftalmológica. Nesse contexto, a análise do custo cirúrgico isolado precisa ser situada em uma perspectiva ampliada de trajetória da doença, na qual o atraso terapêutico pode deslocar o sistema para estágios de maior complexidade clínica e maior custo social acumulado^(11,12)^.

Sob o ponto de vista do acesso, a literatura também tem chamado atenção para o impacto dos atrasos entre a indicação cirúrgica e a entrada efetiva no centro cirúrgico. Estudo multicêntrico recente sobre cirurgias filtrantes de glaucoma identificou tempo mediano de espera de 44 dias entre a indicação e a realização do procedimento, destacando que a etapa pré-operatória frequentemente concentra a maior parcela da demora assistencial^(13)^.

Embora os contextos de organização do cuidado sejam distintos, esse achado dialoga com o presente estudo ao evidenciar que o tempo de espera é um componente concreto do risco clínico e da ineficiência organizacional. Mesmo quando o atraso não se traduz imediatamente em cegueira ou deficiência visual grave, evidências de seguimento de longo prazo indicam maior perda de campo visual e maior velocidade de progressão quando o tratamento é postergado, o que reforça a centralidade do acesso oportuno como desfecho assistencial relevante^(14)^.

A persistência de tempo prolongado de espera para a realização do procedimento reforça que o problema central não se restringe ao custo direto da cirurgia, mas à insuficiência do sistema em assegurar oportunidade terapêutica em tempo clinicamente adequado. No glaucoma, atrasos no tratamento podem resultar em progressão irreversível da perda visual, com repercussões funcionais, sociais e econômicas amplas. Sob essa perspectiva, a fila cirúrgica deixa de ser apenas um indicador administrativo e passa a representar um marcador de risco assistencial, com potencial impacto sobre incapacidade, dependência e necessidade futura de reabilitação e suporte social. A interpretação dos resultados, portanto, deve considerar não apenas o custo do procedimento em si, mas também o custo da não realização tempestiva do cuidado especializado.

No campo da enfermagem perioperatória, os achados assumem especial relevância por evidenciarem que a gestão qualificada de insumos, o preparo seguro do ambiente cirúrgico e a racionalização do giro de sala podem contribuir para maior eficiência sem comprometimento da qualidade assistencial. Em hospitais universitários, onde coexistem assistência, ensino e pesquisa, a previsibilidade do consumo de materiais e medicamentos é estratégica para o planejamento do cuidado e para a redução de cancelamentos evitáveis. Dessa forma, a enfermagem ocupa posição central na tradução de evidências econômicas em práticas organizacionais capazes de ampliar a produtividade cirúrgica e qualificar a resposta institucional à demanda reprimida.

A literatura sobre gestão perioperatória oferece elementos adicionais para interpretar esse cenário. Revisão integrativa brasileira demonstrou que estratégias baseadas em Lean e Six Sigma podem otimizar o fluxo de pacientes, o processo de trabalho e indicadores de desempenho no perioperatório, favorecendo maior estabilidade operacional e identificação de gargalos evitáveis^(15)^.

Em paralelo, revisão sistemática internacional sobre tempo de giro de sala mostrou que medidas mutáveis, como processamento paralelo, padronização do preparo, coordenação entre equipes e reorganização do fluxo, estão consistentemente associadas à melhoria da eficiência do centro cirúrgico^(16)^.

Para a realidade de serviços oftalmológicos, em que o volume de procedimentos pode ser fortemente sensível a atrasos cumulativos, tais achados sugerem que ganhos marginais de organização podem produzir impacto expressivo sobre a capacidade anual de oferta. Nessa direção, métodos de custeio por atividade e suas variações orientadas pelo tempo vêm sendo descritos como ferramentas estratégicas para aproximar análise econômica e gestão do cuidado, ao tornar visíveis os direcionadores reais de custo ao longo da jornada assistencial^(17,18)^.

Em hospitais de ensino, essa abordagem é particularmente útil porque permite distinguir custo inerente ao procedimento, custo associado à ineficiência operacional e custo decorrente da complexidade institucional. Mais do que mensurar gastos, o microcusteio pode apoiar decisões sobre padronização de kits cirúrgicos, rastreamento de consumo, negociação de compras, desenho de processos e priorização de intervenções de melhoria. Para a enfermagem e para a gestão hospitalar, isso significa deslocar o debate do controle meramente financeiro para a produção de valor assistencial, articulando segurança, produtividade e sustentabilidade.

Em termos de gestão da rede, os resultados sugerem que a ampliação da oferta cirúrgica depende menos de revisão imediata do valor de reembolso e mais de estratégias de coordenação assistencial, planejamento de agenda operatória e fortalecimento da regulação. A coexistência entre viabilidade econômica do procedimento e persistência de fila expressiva indica que barreiras organizacionais podem estar produzindo ineficiência alocativa, na medida em que um procedimento potencialmente sustentável não se converte em cobertura suficiente. Esse achado é particularmente relevante para políticas públicas de saúde ocular, pois aponta a necessidade de integrar avaliação econômica, monitoramento de produção e gestão do acesso como dimensões indissociáveis da resposta assistencial ao glaucoma.

Os resultados devem ser interpretados à luz de algumas limitações. A análise concentrou-se em uma única instituição e considerou apenas custos diretos, o que restringe a generalização dos achados e impede estimativa mais abrangente do custo total hospitalar da trabeculectomia. Além disso, a utilização de prontuários físicos e registros secundários pode introduzir perdas informacionais e limita a exploração de variáveis clínicas mais detalhadas. Ainda assim, o estudo oferece evidência empírica relevante ao documentar, com base em dados reais, a distância entre sustentabilidade econômica do procedimento e capacidade concreta de resposta do serviço, aspecto ainda pouco explorado na literatura nacional.

Outra limitação refere-se à ausência de transparência na composição dos componentes utilizados para definição do valor de reembolso da APAC, o que restringe comparações diretas entre os custos identificados por microcusteio e os critérios empregados pelo sistema de financiamento do SUS.

## CONCLUSÃO

Este estudo demonstrou que, no recorte dos custos diretos mensurados, a trabeculectomia realizada em um hospital universitário federal de referência em oftalmologia, localizado na região Sudeste do Brasil, apresentou custo médio inferior ao valor reembolsado pelo Sistema Único de Saúde. Esse resultado indica viabilidade econômico-operacional do procedimento sob a perspectiva dos materiais e medicamentos intraoperatórios, mas não esgota a análise sobre sustentabilidade institucional, uma vez que custos indiretos e estruturais não foram contemplados.

Ao mesmo tempo, a manutenção de fila expressiva para o procedimento evidenciou que a viabilidade econômica do custo direto não se traduz automaticamente em acesso oportuno, apontando para a influência de fatores organizacionais, regulatórios e de capacidade instalada na resposta assistencial ao glaucoma. Os achados reforçam a utilidade do microcusteio como ferramenta de apoio à gestão hospitalar e à enfermagem perioperatória, ao permitir maior visibilidade sobre o consumo de insumos e subsidiar estratégias de planejamento do cuidado, organização do fluxo cirúrgico e qualificação do uso de recursos no contexto do SUS.

## Data Availability

Conforme preconizado pela Ciência Aberta, repositório no qual os dados de pesquisa foram depositados em sua integralidade. Nome do repositório: http://www.repositorio-bc.unirio.br/. URL ou DOI: http://hdl.handle.net/unirio/14982. Data do depósito: 2026-04-02.

# Translation: Costo directo de la trabeculectomía en el Sistema Único de Salud: análisis de microcostos en el hospital universitario*

**DOI:** 10.1590/1980-220X-REEUSP-2026-0293es

**Authors:** Joice Alves Cabral, Priscila de Castro Handem, Ana Paula Rodrigues Siqueira, Giovanni Nicola Umberto Italiano Colombini, Daniel Aragão Machado, Carlos Roberto Lyra da Silva, Alexander Itria, Thiago Louro, Andrea Alves de Andrade, Roberto Carlos Lyra da Silva

**Affiliations:** 1 Universidade Federal do Estado do Rio de Janeiro, Hospital Universitário Gaffrée e Guinle, Rio de Janeiro, RJ, Brasil.; 2 Universidade Federal do Estado do Rio de Janeiro, Escola de Enfermagem Alfredo Pinto, Rio de Janeiro, RJ, Brasil.; 3 Instituto Nacional do Câncer, Programa de Pós-Graduação em Oncologia, Rio de Janeiro, RJ, Brasil.; 4 Universidade Federal de São Carlos, São Carlos, SP, Brasil.; 5 Universidade Federal Fluminense, Rio das Ostras, RJ, Brasil.; 6 Universidade Federal do Rio de Janeiro, Centro de Ciências Matemáticas e da Natureza, Rio de Janeiro, RJ, Brasil.

**Keywords:** Glaucoma, Costos y Análisis de Costo, Procedimientos Quirúrgicos Oftalmológicos, Sistema Único de Salud, Enfermería Perioperatoria.

## Abstract

**Objetivo:**

Estimar el costo directo de la trabeculectomía en un hospital universitario perteneciente al Sistema Único de Salud (SUS) de Brasil y compararlo con el reembolso por dicho procedimiento.

**Método:**

Estudio documental, retrospectivo y cuantitativo, con microcosteo basado en Activity-Based Costing. Se analizaron 67 historias clínicas elegibles procedentes de 76 trabeculectomías realizadas entre 2022 y 2024. Se tuvieron en cuenta los materiales quirúrgicos y los medicamentos administrados durante la intervención.

**Resultados:**

El costo directo promedio fue de R$ 388,32 por procedimiento, mientras que el monto promedio facturado al SUS fue de R$ 602,25 por procedimiento. Los hilos de sutura representaron el 45% del coste total, seguidos de los medicamentos intraoperatorios (28%) y los consumibles (27%). Se observó la persistencia de la lista de espera quirúrgica y un aumento en los tiempos de espera regulados.

**Conclusión:**

La trabeculectomía tuvo un coste directo inferior al reembolso del SUS; sin embargo, la viabilidad económica del procedimiento no se tradujo automáticamente en un acceso oportuno.

## INTRODUCCIÓN

El glaucoma es una neuropatía óptica progresiva, caracterizada por alteraciones estructurales en el disco óptico y en la capa de fibras nerviosas de la retina, frecuentemente asociada a niveles elevados de presión intraocular (PIO). Considerado una causa importante de ceguera irreversible en el mundo, el glaucoma impone una carga social y económica significativa a los sistemas de salud, especialmente en países de ingreso medio, como Brasil^(1,2)^. En el marco del Sistema Único de Salud (SUS), el manejo de esta afección exige un equilibrio entre el diagnóstico precoz, la terapia farmacológica continua y la intervención quirúrgica oportuna^(3)^.

La trabeculectomía (TREC) sigue siendo, desde su introducción, la técnica quirúrgica de «referencia» para el control de la PIO en los casos en que el tratamiento clínico con colirios es insuficiente o inviable. Mediante la creación de una fístula que permite el drenaje del humor acuoso hacia el espacio subconjuntival, la TREC tiene como objetivo estabilizar el campo visual y prevenir la progresión hacia la amaurosis.

A pesar de la aparición de nuevas tecnologías, como los implantes de drenaje y las cirugías mínimamente invasivas, la trabeculectomía sigue siendo relevante por su eficacia a largo plazo y por su potencial de menor costo operativo para el sistema de salud pública^(4)^. Sin embargo, el panorama de la salud pública brasileña se caracteriza por restricciones de financiamiento y por incertidumbres respecto a la suficiencia de los montos reembolsados por el Sistema de Gestión de la Tabla de Procedimientos, Medicamentos, Órtesis, Prótesis y Materiales Especiales del SUS (SIGTAP) para cubrir los costos reales de los insumos, especialmente en los hospitales universitarios, que acumulan funciones de atención, enseñanza e investigación.

La falta de estimaciones precisas del costo directo de los procedimientos compromete las decisiones administrativas y puede limitar la oferta de servicios esenciales. En el caso de la trabeculectomía, conocer la relación entre el costo de los insumos, el reembolso del SUS y el acceso a través del sistema de regulación es fundamental para distinguir los problemas de financiamiento de aquellos relacionados con la organización de la atención y la capacidad productiva del servicio.

Este estudio se diseñó para apoyar la gestión hospitalaria y la enfermería perioperatoria, al generar evidencia empírica sobre el costo directo, la sostenibilidad del procedimiento y las barreras organizativas para el acceso quirúrgico. La pregunta de investigación fue: ¿cuál es el costo directo real de la trabeculectomía realizada? Siendo el escenario un hospital universitario federal de referencia en oftalmología, ubicado en la región sudeste de Brasil, ¿cómo se comporta este valor en relación con el reembolso del SUS y el acceso a través del sistema de regulación?

La Evaluación de Tecnologías en Salud busca ofrecer evidencia para la toma de decisiones sobre la incorporación y el uso de procedimientos. Sin embargo, en el ámbito de la gestión hospitalaria, los métodos de microcosteo siguen siendo esenciales para identificar el consumo por partida por paciente y generar estimaciones más precisas del costo directo de la atención, en contraste con los enfoques agregados de macrocosteo^(5)^.

## MÉTODO

Se trata de un estudio cuantitativo, descriptivo, documental y retrospectivo, con la aplicación del microcosteo desde la perspectiva hospitalaria, basado en el método *Activity-Based Costing* (ABC). El escenario fue un hospital universitario federal de referencia en oftalmología ubicado en la región sudeste de Brasil, dentro del Sistema Único de Salud. El período de estudio abarcó las cirugías realizadas entre 2022 y 2024.

La muestra estuvo compuesta por 67 expedientes médicos con datos completos, provenientes de 76 trabeculectomías identificadas en el período. Se incluyeron pacientes sometidos a trabeculectomía primaria, con registro completo de materiales, medicamentos, tiempo quirúrgico, edad, sexo y valor facturado. Se excluyeron las cirugías combinadas y los expedientes con datos incompletos que impidieran estimar el costo directo del procedimiento.

La recolección de datos se realizó a partir del mapa quirúrgico, los expedientes físicos, las hojas de facturación y los registros institucionales de materiales y medicamentos. Se extrajeron variables demográficas y de atención (edad, sexo y tiempo quirúrgico), variables económicas (insumos utilizados, costo unitario y costo total directo) y variables de facturación (monto reembolsado por el SUS por procedimiento). La unidad monetaria utilizada fue el real brasileño en el año 2025.

Los montos de reembolso considerados en este estudio correspondieron a los montos efectivamente facturados y aprobados por el SUS para cada procedimiento, según lo registrado en las hojas de facturación de los expedientes analizados. De esta manera, los montos presentados reflejan la producción hospitalaria realizada entre 2022 y 2024 y no solo el valor nominal vigente en la tabla SIGTAP para una competencia específica. Aunque el valor nominal que figura en la tabla SIGTAP es fijo por especialidad, los valores analizados corresponden a los montos efectivamente aprobados y registrados en las hojas de facturación de los expedientes médicos, sujetos a las normas de tramitación vigentes en cada período.

El microcosteo se llevó a cabo en tres etapas: identificación de los insumos utilizados en cada cirugía, valoración de los artículos a partir de los registros institucionales de adquisición y comparación de los costos calculados con los montos efectivamente facturados al SUS. Se consideraron los materiales quirúrgicos y los medicamentos intraoperatorios directamente relacionados con la realización de la trabeculectomía. No se incluyeron costos indirectos, como recursos humanos, energía, esterilización, depreciación de equipos y gastos administrativos.

El costo directo total por cirugía se estimó mediante la suma de los costos unitarios multiplicados por las cantidades consumidas en cada procedimiento, según la fórmula: costo directo total = Σ (cantidad del artículo × costo unitario del artículo). Los datos se organizaron en hojas de cálculo y se analizaron mediante estadística descriptiva, con el cálculo de la media, la mediana, la desviación estándar y la distribución anual de los procedimientos, los costos y los reembolsos. Aspectos éticos: El estudio respetó los principios éticos de la Resolución n.º 466/2012, con el uso de datos primarios anonimizados y la aprobación del Comité de Ética en Investigación, mediante el dictamen n.º 7.196.235^(6,7)^.

## RESULTADOS

El análisis de los 67 expedientes médicos elegibles, procedentes de un total de 76 cirugías realizadas entre 2022 y 2024, permitió caracterizar con mayor precisión el perfil asistencial y económico de la trabeculectomía en el contexto investigado.

Se realizaron 17 trabeculectomías en 2022, 35 en 2023 y 24 en 2024. Desde el punto de vista demográfico y clínico, la cohorte presentó una edad promedio de 59,09 años (desviación estándar: 24,87), con una mediana de 66,5 años, lo que evidencia una distribución asimétrica y una concentración de casos en grupos de edad más avanzados, aunque con la presencia de pacientes más jóvenes.

La duración promedio de la cirugía fue de 1 h 46 min (desviación estándar: 37 min 08 s). Los valores anuales mostraron un ligero aumento del costo directo promedio por procedimiento a lo largo de la serie, de R$ 351,73 en 2022 a R$ 394,00 en 2023 y R$ 404,30 en 2024. Los reembolsos totales del SUS se mantuvieron superiores a los costos directos en todos los años analizados, lo que resultó en una diferencia promedio favorable de 213,93 reales por procedimiento. (Tabla 1).

**Tabla 1:** Comparación anual entre el reembolso del SUS y el costo directo de la trabeculectomía – Río de Janeiro, RJ, Brasil, 2022–2024^1^.

Año	Número de procedimientos	Reembolso SUS total (R$)	Costo directo total (R$)	Costo directo por procedimiento (R$)
2022	17	13.149,02	5.979,43	351,73
2023	35	26.725,30	13.789,85	394,00
2024	24	14.929,05	7.681,76	404,30

Fuente: Datos de la investigación.

El desglose del costo directo promedio por procedimiento, estimado en 388,32 reales brasileños, puso de manifiesto una concentración de los gastos en elementos fundamentales para la ejecución técnica segura de la trabeculectomía, con predominio de los hilos de sutura (45,0 %), seguidos por los medicamentos intraoperatorios (28,0 %) y los materiales de consumo (27,0 %). Esta distribución muestra una dependencia de insumos especializados y un margen reducido para la reducción de costos sin que ello afecte la calidad de la atención (Tabla 2).

**Tabla 2:** Composición del costo directo promedio de la trabeculectomía, según categoría de insumo – Río de Janeiro, RJ, Brasil, 2022–2024.

Categoría de insumo	Costo medio (R$)	Representatividad (%)
Hilos de sutura (nailon 10-0 y 8-0)	174,74	45,0
Medicamentos intraoperatorios (mitomicina y anestésicos)	108,73	28,0
Materiales de consumo (bisturís, guantes, gasas y campos)	104,85	27,0
**Total**	**388,32**	**100,0**

Fuente: Datos de la investigación.

Los indicadores de acceso mostraron un desajuste entre el potencial de sostenibilidad del procedimiento y la capacidad de respuesta del sistema. El tiempo promedio de espera en el SISREG aumentó de 18 días, en 2020, a 122 días, en 2025, al tiempo que persistía una demanda reprimida superior a la capacidad anual de atención quirúrgica del servicio.

## DISCUSIÓN

Los resultados de este estudio ofrecen una interpretación relevante para el debate sobre la sostenibilidad de la atención quirúrgica del glaucoma en el marco del Sistema Único de Salud. La identificación de un costo directo promedio inferior al valor reembolsado por el SUS sugiere, en principio, la viabilidad económica de la trabeculectomía desde la perspectiva de los insumos medidos, en consonancia con los análisis económicos ya aplicados al campo del glaucoma^(4,7)^.

Estudios previos sobre el glaucoma indican que la trabeculectomía sigue siendo un procedimiento clínico muy eficaz y ampliamente utilizado, especialmente en entornos con recursos limitados. Además, las nuevas tecnologías quirúrgicas, si bien representan alternativas terapéuticas prometedoras para casos seleccionados, pueden tener costos más elevados y una disponibilidad más limitada en los sistemas de salud pública. En este contexto, la trabeculectomía sigue siendo una alternativa relevante para la sostenibilidad de los servicios de salud.

Sin embargo, este hallazgo no permite una interpretación simplificada de la suficiencia del financiamiento, ya que el microcosto adoptado contempló exclusivamente materiales y medicamentos intraoperatorios, sin incluir componentes indirectos y estructurales que intervienen en la prestación de la atención^(5)^. Por lo tanto, la principal contribución analítica de este estudio radica en demostrar que la relación entre costo y reembolso debe interpretarse en relación con la capacidad instalada, la organización del proceso de trabajo y la eficacia del acceso quirúrgico. Cabe destacar que la comparación se realizó con base en los montos efectivamente facturados por la institución durante el período estudiado, y no exclusivamente en los montos nominales vigentes en un único período de la tabla SIGTAP, lo cual debe tenerse en cuenta al interpretar los resultados.

La literatura internacional reciente refuerza la pertinencia de esta interpretación. Un modelo económico derivado del *Treatment of Advanced Glaucoma Study* demostró que, en pacientes con glaucoma primario de ángulo abierto avanzado, la trabeculectomía presenta una mayor probabilidad de ser rentable a lo largo de la vida en comparación con el tratamiento exclusivamente farmacológico, con una ganancia incremental en la calidad de vida y una relación costo-QALY considerada aceptable en los sistemas de salud públicos^(8)^.

Aunque este tipo de análisis se basa en un horizonte temporal y una estructura de costos diferentes a los empleados en el presente estudio, sus resultados coinciden al sostener que la cirugía filtrante no debe interpretarse únicamente como un incremento del gasto inmediato, sino como una intervención potencialmente capaz de reducir la progresión de la enfermedad, la necesidad de múltiples terapias y los costos futuros asociados a la discapacidad visual.

Este debate cobra aún mayor relevancia cuando se considera la carga clínica, social y económica del glaucoma. Estudios recientes muestran que la progresión de la enfermedad está asociada con un deterioro de la calidad de vida, un deterioro funcional, una mayor frecuencia de ansiedad y depresión, y un incremento de los costos directos e indirectos a medida que la pérdida de la visión se agrava^(9,10)^.

Las actualizaciones sobre la carga global de la enfermedad también indican que el glaucoma sigue figurando entre las causas más relevantes de años vividos con discapacidad relacionados con la visión, especialmente en poblaciones de edad avanzada y en contextos de desigualdad en el acceso a la atención oftalmológica. En este contexto, el análisis del costo quirúrgico aislado debe situarse en una perspectiva más amplia de la trayectoria de la enfermedad, en la que el retraso terapéutico puede llevar al sistema a etapas de mayor complejidad clínica y mayor costo social acumulado^(11,12)^.

Desde el punto de vista del acceso, la literatura también ha llamado la atención sobre el impacto de las demoras entre la indicación quirúrgica y el ingreso efectivo al quirófano. Un estudio multicéntrico reciente sobre cirugías de filtración para el glaucoma identificó un tiempo mediano de espera de 44 días entre la indicación y la realización del procedimiento, destacando que la etapa preoperatoria suele concentrar la mayor parte de la demora en la atención^(13)^.

Aunque los contextos de organización de la atención son distintos, este hallazgo concuerda con el presente estudio al evidenciar que el tiempo de espera es un componente concreto del riesgo clínico y de la ineficiencia organizacional. Incluso cuando el retraso no se traduce inmediatamente en ceguera o discapacidad visual grave, la evidencia de seguimiento a largo plazo indica una mayor pérdida del campo visual y una mayor velocidad de progresión cuando se pospone el tratamiento, lo que refuerza la importancia fundamental del acceso oportuno como resultado relevante de la atención^(14)^.

La persistencia de tiempos de espera prolongados para realizar el procedimiento refuerza la idea de que el problema central no se limita al costo directo de la cirugía, sino a la insuficiencia del sistema para garantizar la oportunidad terapéutica en un plazo clínicamente adecuado. En el glaucoma, los retrasos en el tratamiento pueden resultar en una progresión irreversible de la pérdida visual, con amplias repercusiones funcionales, sociales y económicas. Desde esta perspectiva, la lista de espera quirúrgica deja de ser solo un indicador administrativo y pasa a representar un marcador de riesgo asistencial, con un impacto potencial sobre la discapacidad, la dependencia y la necesidad futura de rehabilitación y apoyo social. La interpretación de los resultados, por lo tanto, debe considerar no solo el costo del procedimiento en sí, sino también el costo de no brindar oportunamente la atención especializada.

En el ámbito de la enfermería perioperatoria, los hallazgos cobran especial relevancia al evidenciar que la gestión calificada de los insumos, la preparación segura del entorno quirúrgico y la racionalización del ciclo de uso de los quirófanos pueden contribuir a una mayor eficiencia sin comprometer la calidad de la atención. En los hospitales universitarios, donde coexisten la atención, la enseñanza y la investigación, la previsibilidad del consumo de materiales y medicamentos es estratégica para la planificación de la atención y para la reducción de cancelaciones evitables. De esta manera, la enfermería ocupa un lugar central en la traducción de la evidencia económica en prácticas organizativas capaces de ampliar la productividad quirúrgica y mejorar la respuesta institucional a la demanda reprimida.

La literatura sobre gestión perioperatoria ofrece elementos adicionales para interpretar este escenario. Una revisión integrativa brasileña demostró que las estrategias basadas en Lean y Six Sigma pueden optimizar el flujo de pacientes, el proceso de trabajo y los indicadores de desempeño en el ámbito perioperatorio, lo que favorece una mayor estabilidad operativa y la identificación de cuellos de botella evitables^(15)^.

Paralelamente, una revisión sistemática internacional sobre el tiempo de rotación de los quirófanos mostró que medidas modificables, como el procesamiento paralelo, la estandarización de la preparación, la coordinación entre equipos y la reorganización del flujo, se asocian de manera consistente con la mejora de la eficiencia del quirófano^(16)^.

En el contexto de los servicios oftalmológicos, donde el volumen de procedimientos puede ser muy sensible a los retrasos acumulativos, estos hallazgos sugieren que las mejoras marginales en la organización pueden tener un impacto significativo en la capacidad de atención anual. En este sentido, los métodos de costeo por actividad y sus variantes orientadas al tiempo se han descrito como herramientas estratégicas para acercar el análisis económico y la gestión de la atención, al hacer visibles los verdaderos factores que determinan los costos a lo largo del proceso de atención^(17,18)^.

En los hospitales de enseñanza, este enfoque resulta particularmente útil porque permite distinguir entre el costo inherente al procedimiento, el costo asociado a la ineficiencia operativa y el costo derivado de la complejidad institucional. Más allá de medir los gastos, el microcosteo puede respaldar decisiones sobre la estandarización de kits quirúrgicos, el seguimiento del consumo, la negociación de compras, el diseño de procesos y la priorización de intervenciones de mejora. Para el personal de enfermería y la administración hospitalaria, esto significa desplazar el debate del control meramente financiero hacia la generación de valor asistencial, articulando seguridad, productividad y sostenibilidad.

En términos de gestión de la red, los resultados sugieren que la ampliación de la oferta quirúrgica depende menos de una revisión inmediata del valor de reembolso y más de estrategias de coordinación asistencial, planificación del calendario quirúrgico y fortalecimiento de la regulación. La coexistencia entre la viabilidad económica del procedimiento y la persistencia de una lista de espera significativa indica que las barreras organizativas podrían estar generando ineficiencia en la asignación de recursos, en la medida en que un procedimiento potencialmente sostenible no se traduce en una cobertura suficiente. Este hallazgo es particularmente relevante para las políticas públicas de salud ocular, ya que señala la necesidad de integrar la evaluación económica, el monitoreo de la producción y la gestión del acceso como dimensiones inseparables de la respuesta asistencial al glaucoma.

Los resultados deben interpretarse a la luz de algunas limitaciones. El análisis se centró en una sola institución y consideró únicamente los costos directos, lo que restringe la generalización de los hallazgos e impide una estimación más amplia del costo hospitalario total de la trabeculectomía. Además, el uso de expedientes médicos físicos y registros secundarios puede introducir pérdidas de información y limita el análisis de variables clínicas más detalladas. Aun así, el estudio ofrece evidencia empírica relevante al documentar, con base en datos reales, la brecha entre la sostenibilidad económica del procedimiento y la capacidad concreta de respuesta del servicio, un aspecto aún poco explorado en la literatura nacional.

Otra limitación se refiere a la falta de transparencia en la composición de los componentes utilizados para definir el valor de reembolso de la APAC (Autorización de procedimientos de alta complejidad), lo que restringe las comparaciones directas entre los costos identificados mediante el microcosteo y los criterios empleados por el sistema de financiamiento del SUS.

## CONCLUSIÓN

Este estudio demostró que, en lo que respecta a los costos directos medidos, la trabeculectomía realizada en un hospital universitario federal de referencia en oftalmología, ubicado en la región sudeste de Brasil, presentó un costo promedio inferior al valor reembolsado por el Sistema Único de Salud. Este resultado indica la viabilidad económica y operativa del procedimiento desde la perspectiva de los materiales y medicamentos intraoperatorios, pero no agota el análisis sobre la sostenibilidad institucional, ya que no se contemplaron los costos indirectos y estructurales.

Al mismo tiempo, el mantenimiento de una lista de espera significativa para el procedimiento puso de manifiesto que la viabilidad económica del costo directo no se traduce automáticamente en un acceso oportuno, lo que apunta a la influencia de factores organizacionales, normativos y de capacidad instalada en la atención del glaucoma. Los hallazgos refuerzan la utilidad del microcosteo como herramienta de apoyo a la gestión hospitalaria y a la enfermería perioperatoria, al permitir una mayor visibilidad sobre el consumo de insumos y respaldar estrategias de planificación de la atención, organización del flujo quirúrgico y optimización del uso de recursos en el contexto del SUS.

## Data Availability

De conformidad con los principios de la Ciencia Abierta, se indica el repositorio en el que se han depositado los datos de la investigación en su totalidad. Nombre del repositorio: http://www.repositorio-bc.unirio.br/. URL o DOI: http://hdl.handle.net/unirio/14982. Fecha de depósito: 2 de abril de 2026.
